# Prevalence and Clonal Distribution of Azole-Resistant *Candida parapsilosis* Isolates Causing Bloodstream Infections in a Large Italian Hospital

**DOI:** 10.3389/fcimb.2020.00232

**Published:** 2020-05-25

**Authors:** Cecilia Martini, Riccardo Torelli, Theun de Groot, Elena De Carolis, Grazia Angela Morandotti, Giulia De Angelis, Brunella Posteraro, Jacques F. Meis, Maurizio Sanguinetti

**Affiliations:** ^1^Dipartimento di Scienze Biotecnologiche di base, Cliniche Intensivologiche e Perioperatorie, Università Cattolica del Sacro Cuore, Rome, Italy; ^2^Dipartimento di Scienze di Laboratorio e Infettivologiche, Fondazione Policlinico Universitario A. Gemelli IRCCS, Rome, Italy; ^3^Department of Medical Microbiology and Infectious Diseases, Canisius Wilhelmina Hospital (CWZ), Nijmegen, Netherlands; ^4^Dipartimento di Scienze Gastroenterologiche, Endocrino-Metaboliche e Nefro-Urologiche, Fondazione Policlinico Universitario A. Gemelli IRCCS, Rome, Italy; ^5^Centre of Expertise in Mycology Radboudumc/Canisius Wilhelmina Hospital, Nijmegen, Netherlands; ^6^Bioprocess Engineering and Biotechnology Graduate Program, Federal University of Paraná, Curitiba, Brazil

**Keywords:** bloodstream infections, *Candida parapsilosis*, antifungal susceptibility, azole resistance, microsatellite genotyping, molecular clones

## Abstract

The most prevalent cause of nosocomial bloodstream infection (BSI) among non-*C. albicans Candida* species, *Candida parapsilosis*, may not only be resistant to azole antifungal agents but also disseminate to vulnerable patients. In this survey of BSIs occurring at a large Italian hospital between May 2014 and May 2019, *C. parapsilosis* accounted for 28.5% (241/844) of all *Candida* isolates causing BSI episodes. The majority of episodes (151/844) occurred in medical wards. Across the 5 yearly periods, the rates of azole non-susceptibility were 11.8% (4/34), 17.8% (8/45), 28.6% (12/42), 32.8% (19/58), and 17.7% (11/62), respectively, using the Sensititre YeastOne® method. Among azole non-susceptible isolates (54/241; 22.4%), 49 were available for further investigation. Using the CLSI reference method, all 49 isolates were resistant to fluconazole and, except one (susceptible dose-dependent), to voriconazole. Forty (81.6%) isolates harbored the Erg11p Y132F substitution and nine (18.4%) isolates the Y132F in combination with the Erg11p R398I substitution. According to their genotypes, as defined using a microsatellite analysis based on six short tandem repeat markers, 87.7% of isolates (43/49) grouped in two major clusters (II and III), whereas 4.1% of isolates (2/49) belonged to a separate cluster (I). Interestingly, all the isolates from cluster II harbored the Y132F substitution, and those from cluster III harbored both Y132F and R398I substitutions. Of 56 non-Italian isolates included as controls, two Indian isolates with the Y132F substitution had a genotype clearly differing from that of the isolates from clusters II and I. In conclusion, these findings show the dominance of clonal Y132F isolates in our hospital and suggest detection of the Y132F substitution as helpful tool to prevent transmission among hospitalized patients at risk of BSI.

## Introduction

Among non-*C. albicans Candida* (NCAC) species (Miceli et al., 2011), *Candida parapsilosis* has emerged as a common cause of bloodstream infection (BSI), which represents—together with other forms of invasive candidiasis (i.e., non-candidemia forms)—the most prevalent systemic fungal nosocomial infection (Kullberg and Arendrup, 2015; Mesini et al., 2020). Although the prevalence of NCAC species may vary considerably depending on geographical location (Lamoth et al., 2018), *C. parapsilosis* remains the second or third (after *Candida albicans*) most frequently isolated *Candida* species from hospitalized patients globally (Guinea, 2014). For instance, a 9-year hospital laboratory-based survey of BSI cases published in 2015 (Posteraro et al., 2015) showed that *C. parapsilosis* complex—including *C. parapsilosis* and its two closely related species *Candida metapsilosis* and *Candida orthopsilosis* (Tavanti et al., 2005)—accounted for ~21% (262/1,250) of all *Candida*-driven and non-*Candida*-driven BSIs (second most common after *C. albicans*). Similarly, Govender et al. (2016) surveyed 2,172 cases of candidemia from a 19-month period in public sector or private-sector hospitals across South Africa, showing that *C. parapsilosis* was responsible for 35% of public-sector cases (second most common after *C. albicans*) and >50% in the private sector (outranking *C. albicans*).

While *C. parapsilosis* BSIs generally result in lower morbidity and mortality rates than *C. albicans* BSIs (Tóth et al., 2019), the dissemination of *C. parapsilosis* from healthcare environments to vulnerable patients is troubling (Lamoth et al., 2018), as is the increasing resistance to azole antifungal drugs (Thomaz et al., 2018; Pristov and Ghannoum, 2019). Only 199 (37%) of 531 *C. parapsilosis* isolates in the survey by Govender et al. (2016) were susceptible to the triazoles fluconazole and voriconazole, whereas only 18 (7%) of 262 *C. parapsilosis* isolates in the survey by Posteraro et al. (2015) were non-susceptible [12 susceptible dose-dependent (SDD) and six resistant] to fluconazole. Furthermore, two (0.8%) of 262 isolates in the study by Posteraro et al. (2015) were non-susceptible (all SDD) to voriconazole, whereas 123 (44%) of 282 fluconazole-resistant isolates in the study by Govender et al. (2016) were also resistant to voriconazole. Altogether, these findings suggest that triazole non-susceptible *C. parapsilosis* could significantly affect the management of patients with candidemia (Pappas et al., 2016) by limiting the choice of antifungal agents (Eschenauer et al., 2015).

Unlike other NCAC species, *C. parapsilosis* has historically been associated with neonates (Pammi et al., 2013) and the use of high-glucose containing parenteral nutrition (Pereira et al., 2015), which strongly correlates with the species's abilities of colonizing implanted medical devices (e.g., intravascular catheters) and, consequently, thriving in certain clinical settings (Asadzadeh et al., 2019). The adherence of *C. parapsilosis* to both abiotic and biotic surfaces is a key stage for host colonization and/or biofilm formation (Priest and Lorenz, 2015). This implies that the matrix beta-glucans in biofilms formed by *C. parapsilosis*—the second in producing biofilm among *Candida* species (Soldini et al., 2018)—would sequester multiple types of antifungals (i.e., amphotericin B and fluconazole), hence contributing to the persistence of antifungal-recalcitrant *C. parapsilosis* infections (Pristov and Ghannoum, 2019). Interfering with beta-glucan synthesis, echinocandins (lipopeptidic antifungals) would represent the practical answer for *C. albicans* and NCAC isolates that are resistant to azoles (Whaley et al., 2017). However, prolonged exposure to echinocandins may enable *C. parapsilosis* to not only develop antifungal resistance (Papp et al., 2018) but to cross-infect several patients—perhaps through healthcare workers via hand carriage—hence leading to its high isolation rate (van Asbeck et al., 2009).

As the epidemiology of *C. parapsilosis* is more complex than that might be expected from its pathobiology (Priest and Lorenz, 2015), it has become essential to determine the genetic relatedness among *C. parapsilosis* isolates when clusters appear (van Asbeck et al., 2009). It should also be essential to investigate clustered *C. parapsilosis* isolates for the presence of point mutations in the *ERG11* gene when azole resistance appears (Pristov and Ghannoum, 2019). Until recently, data documented the Y132F amino acid substitution in ERG11p—the *ERG11*-encoded enzyme lanosterol 14-α-demethylase—as the prevalent fluconazole resistance mechanism in isolates (mainly bloodstream isolates) of *C. parapsilosis* worldwide (Grossman et al., 2015; Asadzadeh et al., 2017; Choi et al., 2018; Magobo et al., 2020). Surprisingly, an Indian multicenter laboratory-based surveillance study revealed that in many centers in Delhi and a single center in South India, the K143R amino acid substitution was most prevalent as a single resistance mechanism in either bloodstream or non-bloodstream isolates of *C. parapsilosis* (Singh et al., 2019).

Here we report a retrospective analysis of azole-resistant *C. parapsilosis* isolates, recovered from BSIs over a 5-year period in a large Italian university hospital, aimed at defining their prevalence, their underlying molecular mechanism, and their clonal distribution among different hospital wards.

## Materials and Methods

### Study Setting and Clinical Isolates

The study was retrospectively conducted at the Policlinico Universitario A. Gemelli IRCCS of Rome—a 1,500-bed tertiary care university teaching hospital—using *C. parapsilosis* isolates consecutively collected from blood cultures of patients, who were diagnosed with fungal BSI between May 2014 and May 2019 and were retrieved through a search of the clinical microbiology laboratory information system. Isolates were from single episodes of BSI, and were kept frozen in glycerol and subcultured at the time of this study. Before testing, isolates were identified to the species level (i.e., as *C. parapsilosis sensu stricto*, hereafter referred to as *C. parapsilosis*) by matrix-assisted laser desorption/ionization time-of flight mass spectrometry as previously described (De Carolis et al., 2014). Another 56 *C. parapsilosis* control isolates were from hospitalized patients in non-Italian countries, namely Austria (*n* = 8), Brazil (*n* = 8), Greece (*n* = 8), India (*n* = 8), Iran (*n* = 8), Kuwait (*n* = 8), and the Netherlands (*n* = 8). As shown in Table S1, we provided detailed information for all the isolates, whereas the measured antifungal minimum inhibitory concentration (MIC) values were not available except for Italian isolates (see below).

The study did not require oversight by the institutional ethics committee because the descriptive nature implied only samples that were obtained during routine laboratory activity.

### Antifungal Susceptibility Testing

For all the *C. parapsilosis* isolates included in this study, MIC values were determined against amphotericin B, fluconazole, itraconazole, posaconazole, voriconazole, anidulafungin, caspofungin, micafungin, and flucytosine, as part of routine patient care (Posteraro et al., 2015). The antifungal testing was performed using an adaptation of the Clinical Laboratory Standards Institute (CLSI) method based on the M27-A3 document (CLSI, 2008), namely the Sensititre YeastOne® (SYO) method (Thermo Fisher Scientific, Cleveland, OH, USA). We obtained MIC values by visual inspection of the SYO microdilution broth panels following 24 h of incubation at 35°C, and we calculated MIC_50_, MIC_90_, and ranges using GraphPad Prism 7. We interpreted MIC results according to clinical breakpoints (CBPs) reported in the CLSI M27-S4 document (CLSI, 2012). In particular, for fluconazole, isolates with MIC ≥8 μg/mL were defined as resistant and those with MIC of 4 μg/mL as SDD; for voriconazole, isolates with MIC of ≥1 μg/mL were defined as resistant and those with MIC of 0.25–0.5 μg/mL as SDD. Additionally, we applied the 24-h epidemiological cutoff values (ECVs) of amphotericin B, triazoles (fluconazole, itraconazole, posaconazole, voriconazole), echinocandins (anidulafungin, caspofungin, and micafungin), and flucytosine established for the SYO method and *C. parapsilosis* or other five *Candida* species (Cantón et al., 2012, 2013). Subsequently, the isolates that were either non-susceptible (resistant or SDD/intermediate) or that had non-wild-type (NWT) susceptibility (MIC > ECV) to one or more of the azoles were rechecked for antifungal susceptibility against only fluconazole and voriconazole using the above-mentioned CLSI method, with the two azole antifungals prepared and used as reported in the M27-S4 document (CLSI, 2012).

### DNA Isolation

Genomic DNA from *C. parapsilosis* isolates was obtained with material/equipment of Roche Diagnostics GmbH (Mannheim, Germany). Briefly, we suspended each isolate in 400-μL MagNA Pure Bacteria Lysis Buffer in a tube prefilled with ceramic beads (MagNA Lyser Green Beads), and we used the MagNA Lyser instrument for 30 s at 6,500 rpm to lyse fungal cells mechanically. DNA was then extracted and purified using the MagNA Pure LC instrument with the MagNA Pure DNA isolation kit III (bacteria, fungi), according to the recommendations of the manufacturer.

### *ERG11* Gene Amplification and Sequencing

The entire open reading frame of the *ERG11* gene was sequenced for all *C. parapsilosis* isolates with elevated MICs (i.e., in the resistant or SDD categories) of fluconazole, as well as for the Italian fluconazole-susceptible isolates included in the multilocus microsatellite typing analysis (see below). To this end, we used genomic DNA obtained as above described for PCR amplification with a pair of primers specifically designed by Souza et al. (2015) and shown in Table 1. The resulting PCR products were purified with the MinElute PCR Purification Kit (QIAGEN, Hilden, Germany) and sequenced using an ABI PRISM 3130xl Genetic Analyzer instrument (Applied Biosystems, Palo Alto, CA, USA). DNA sequences were analyzed with the Chromas (http://www.technelysium.com/) and MEGA 6.06 (Tamura et al., 2013) software packages.

**Table 1:** Overview of oligonucleotides used as primers in amplification-based methods.

**Method**	**Target**	**Sequences (5^**′**^-3^**′**^) of oligonucleotides^a^ used as:**	
		**Forward primer**	**Reverse primer**
*ERG11* sequencing	Open reading frame (ORF)		
	Portion 1	CGAGATAATCATCAACGAACATTC	CGTTTAAAACATCCAAAGACCTTA
	Portion 2	AATCTGAGGGTTTCCTTGATGGT	AAAGACCGCATTGACTACCGAT
Microsatellite typing^b^	Short tandem repeat (STR)		
	CTT	FAM-CCTGGCTTGCAATTTCATTT	GCCTCATCGGTGGTGGAATTA
	TTG	HEX-TTGGAGTAACAAGCGCAGAA	GTCGCTTGGACAACTGGTGTA
	ACA	TAMRA-CAATAGCAGCAATGGAGCAG	GTGCTTTTGGTTTGTCCTTGG
	GCTTTT	FAM-CCAGGTTGGACTATCACTG	GGTTTCATTTTGTTGTGAAAA
	GTGTTA	HEX-CCCTTTCAAAAGAAACAGACA	GTTCTATAGATAAAACACACCCCATACA
	TGTTGG	TAMRA-TGGCGTTAGTATTGGCGTTA	GATTGTATCACGCGGGAACTC

a *Sequences of the primer pairs used for ERG11 sequencing or microsatellite typing were described in references of Souza et al. (2015) and Diab-Elschahawi et al. (2012), respectively*.

b *One of the amplification primers (i.e., the forward primer) was labeled with either 6-FAM™, JOE™, or TAMRA™*.

### Microsatellite Typing

We performed multilocus microsatellite typing using a previously described panel of six short tandem repeat (STR) markers, which consisted of three trinucleotide and three hexanucleotide markers, as described by Diab-Elschahawi et al. (2012). One of the amplification primers in each STR panel was labeled with either 6-FAM™, JOE™, or TAMRA™ (https://www.sigmaaldrich.com/), as shown in Table 1. Two multiplex PCRs were carried out in a final volume of 25 μL containing ~1 ng genomic DNA (see above) as described previously (Diab-Elschahawi et al., 2012). The resulting PCR products were analyzed on an automatic sequencer ABI 3500XL Genetic Analyzer (Applied Biosystems). Copy number of the STR markers of *C. parapsilosis* isolates were determined using GeneMapper Software 5 (Applied Biosystems), and the combination of allele numbers for all six diploid loci allowed to define an arbitrary microsatellite genotype (Asadzadeh et al., 2019). Then, a dendrogram was constructed and the genetic relationship of the isolates was determined using the minimum-spanning tree available in the Applied Maths BioNumerics software 7.6.1 (https://www.applied-maths.com/). According to Asadzadeh et al. (2019), we considered the isolates possessing the same alleles for all six diploid loci as having the same genotype (cluster). Genotypes that differed in only one of six diploid loci were closely related genotypes. The reference *C. parapsilosis* strain CBS6318 (a skin isolate) was included in each run to ensure the reproducibility of the STR analysis.

## Results

### Distribution of Isolates Causing BSI

**Figure 1:**
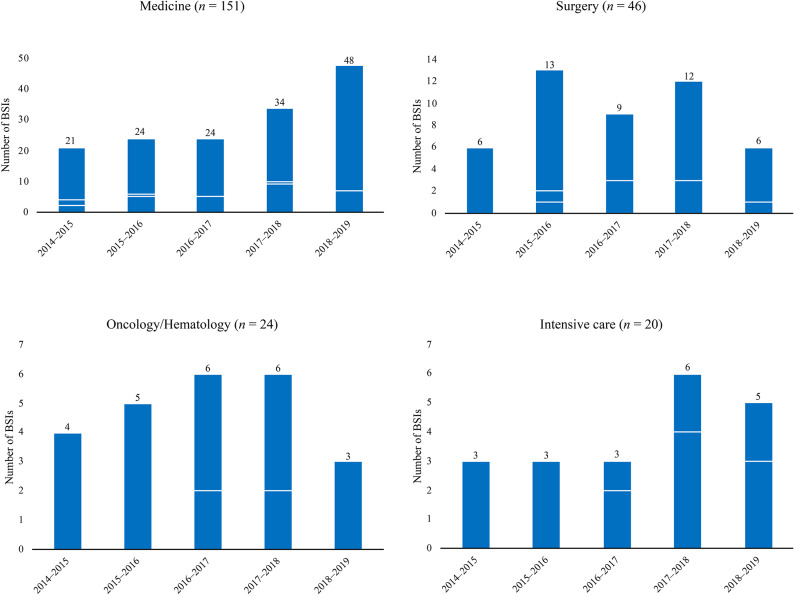
Temporal distributions of *Candida parapsilosis* bloodstream infections (BSIs) in different hospital wards by all (total bar height) and fluconazole or voriconazole non-susceptible (white lines) causative isolates. Overall, BSIs caused by all isolates were 241, whereas those caused by fluconazole non-susceptible isolates were 54 and those caused by voriconazole non-susceptible isolates were 49 (if considering one antifungal drug regardless of the other). As voriconazole non-susceptible isolates were also fluconazole non-susceptible (*n* = 49), the numbers of voriconazole non-susceptible isolates were coincident (one white-line indicated) or lower (the bottom white-line indicated) than the numbers of fluconazole non-susceptible isolates.

Isolates of *C. parapsilosis* were from 241 episodes of BSIs that occurred during the 5-year study period (May 2014–May 2019) in our Italian hospital. Of these cases, 86 were central venous catheter-related BSIs. Overall, 151 (62.7%) were from patients in medical (non-surgical) wards, 46 (19.1%) from patients in surgical wards, 24 (9.9%) from patients in oncology/hematology wards, and 20 (8.3%) from patients in intensive care units (Figure 1). Two hundred twenty patients were adult and 21 patients were pediatric/neonates. Twenty-three patients had multiple episodes of BSI (defined as episodes due to the same fungal species that occurred at least >21 days after the first detected episode). Expectedly (Posteraro et al., 2015), the *C. parapsilosis* isolates represented the 28.5% of all the *Candida* isolates associated with BSI episodes (*n* = 844), of which 14.1% (34/241) in 2014–2015, 18.7% (45/241) in 2015–2016, 17.4% (42/241) in 2016–2017, 24.1% (58/241) in 2017–2018, and 25.7% (62/241) in 2018–2019.

### Azole Antifungal Susceptibilities and *ERG11* Gene Sequences

The MIC results routinely obtained with the SYO method showed that 53 (22.0%) of 241 *C. parapsilosis* isolates were resistant (MICs, ≥8 μg/mL; range, 8–128 μg/mL) and one (0.4%) was SDD to fluconazole (MIC = 4 μg/mL), while 27 (11.2%) were resistant (MICs, ≥1 μg/mL; range, 1–2 μg/mL) and 22 (9.1%) were SDD (MICs, 0.25–0.5 μg/mL; range, 0.25–0.5 μg/mL) to voriconazole, according to the CLSI CBPs (CLSI, 2012). None of remaining 187 isolates showed non-susceptibility or NWT phenotypes to any other antifungal agents tested (data not shown).

Figure 1 depicts the distribution of *C. parapsilosis* BSIs in different wards according to the non-susceptibility (i.e., dose-dependent susceptibility or resistance) to fluconazole and voriconazole of all BSI causative isolates. As shown, the numbers of azole non-susceptible isolates increased across the study's periods, reaching the maximum between May 2017 and May 2018 (*n* = 19). However, this was evident in medical wards (10/34) but not in surgery (3/12), oncology/hematology (2/6), or intensive care wards (4/6). Accordingly, the rates of azole non-susceptibility were 11.8% (4/34), 17.8% (8/45), 28.6% (12/42), 32.8% (19/58), and 17.7% (11/62), respectively.

Table 2 summarizes the antifungal susceptibility profiles for 49 of 54 azole non-susceptible isolates based on SYO testing. Five isolates were not included because they were no longer available. As shown, all 49 isolates were either resistant to fluconazole or, except one, non-susceptible to voriconazole (26 resistant and 22 SDD), according to the interpretive CLSI breakpoints. Then, we retested the 49 isolates against fluconazole and voriconazole using the CLSI reference method. While differences between SYO and CLSI MICs were always within one/two 2-fold microdilutions (as expected), the CLSI reference testing confirmed all 49 isolates to be resistant to fluconazole, with 48 of them also being cross-resistant to voriconazole. Indeed, only one isolate (MIC, 0.25 μg/mL) remained in the SDD category, whereas 22 isolates (MICs, 1 μg/mL) shifted to the resistant category when retested against voriconazole with the CLSI reference method. Consistently, applying the SYO specific epidemiological cutoff values, all 49 isolates appeared NWT against both fluconazole and voriconazole (Table 2), which suggested acquisition of resistance mechanisms in the isolates.

**Table 2:** Antifungal susceptibility results for 49 *Candida parapsilosis* isolates tested with the Sensititre YeastOne® method.

**Antifungal agent**	**No. of isolates**^a^ **with MIC (μg/mL) of:**														**Categories according to clinical breakpoints**^b^			**Categories according to epidemiological cutoffs**^b^	
	**≤0.008**	**0.015**	**0.03**	**0.06**	**0.12**	**0.25**	**0.5**	**1**	**2**	**4**	**8**	**16**	**32**	**≥64**	**Susceptible**	**Susceptible dose-dependent or intermediate**	**Resistant**	**Wild-type**	**Non-wild-type**
AMB						22	24	3							–	–	–	49 (100)	
FLZ											1		4	44	–	–	49 (100)		49 (100)
ITZ			11	**17**	18	3									–	–	–	49 (100)	
PSZ	12		**15**	16	6										–	–	–	49 (100)	
VRZ					1	8	14	**21**	5						1 (2.0)	22 (44.9)	26 (53.1)		49 (100)
ANF						2	**26**	21							49 (100)	–	–	49 (100)	
CSF						17	**27**	5							49 (100)	–	–	49 (100)	
MCF							10	38	1						49 (100)	–	–	49 (100)	
FLC				**42**	6	1									–	–	–	49 (100)	

*AMB, amphotericin B; FLZ, fluconazole; ITZ, itraconazole; PSZ, posaconazole; VRZ, voriconazole; ANF, anidulafungin; CSF, caspofungin; MCF, micafungin; FLC, flucytosine. Bold and underline indicates MIC_50_ and MIC_90_ values, respectively*.

a *Excludes isolates not saved for later use (n = 2) or not providing growth when recovered from their frozen stocks (n = 3). MIC results were interpreted according to clinical breakpoints (all antifungals excluding AMB, ITZ, PSZ, and FLC) and to epidemiological cutoffs (all antifungals). When the isolates were retested with the Clinical and Laboratory Standards Institute reference method for only FLZ and VRZ, MIC results confirmed 49 isolates as FLZ resistant and 48 isolates as VRZ resistant. One isolate remained classified as VRZ susceptible dose-dependent*.

b *Results shown as n (%)*.

Therefore, we characterized the 49 isolates for the presence or absence of point mutation(s) in the *ERG11* gene that translated to amino acid substitution(s) in the Erg11p. Of these isolates, 40 harbored the Y132F substitution and nine the Y132F in combination with the R398I substitution. As shown in Table S1, of 56 additional (non-Italian) isolates studied, two isolates (from India) were resistant to fluconazole and harbored the Y132F substitution. Of note, all Y132F mutations found in Italian isolates were due to the same nucleotide substitution (A395T), whereas searching for other point mutations showed that all 49 Y132F isolates harbored a synonymous substitution (I197I). Additionally, none of the fluconazole-susceptible Italian isolates tested had the Y132F or R398I substitution (Table S1).

### Microsatellite Analysis of Azole-Resistant Isolates

A panel of six STR markers for the microsatellite-based typing allowed the identification of 68 genotypes among 115 *C. parapsilosis* isolates, which included 58 Italian isolates (49 fluconazole-resistant and 9 fluconazole-susceptible), 56 non-Italian isolates (4 fluconazole-resistant and 52 fluconazole-susceptible), and 1 reference strain (CBS6318; fluconazole-susceptible) (Table S1). According to their genotypes, almost all Italian isolates formed three clusters (≥2 identical genotypes at all loci) with two (cluster I), 35 (cluster II), and eight (cluster III) isolates, which were recovered from 2, 34, and 7 patients, respectively. Four isolates were recovered from two patients (2 in cluster II and 2 in cluster III, respectively) during their two recurrences of BSI episodes (the first and second episodes in both patients were 1-year elapsed from each other). Sixty-one genotypes, including the remaining 13 Italian isolates and 49 of 56 non-Italian isolates, involved only one patient each. Three other genotypes involved patients from Greece (three isolates), India (two isolates), and The Netherlands (two isolates), respectively.

**Figure 2:**
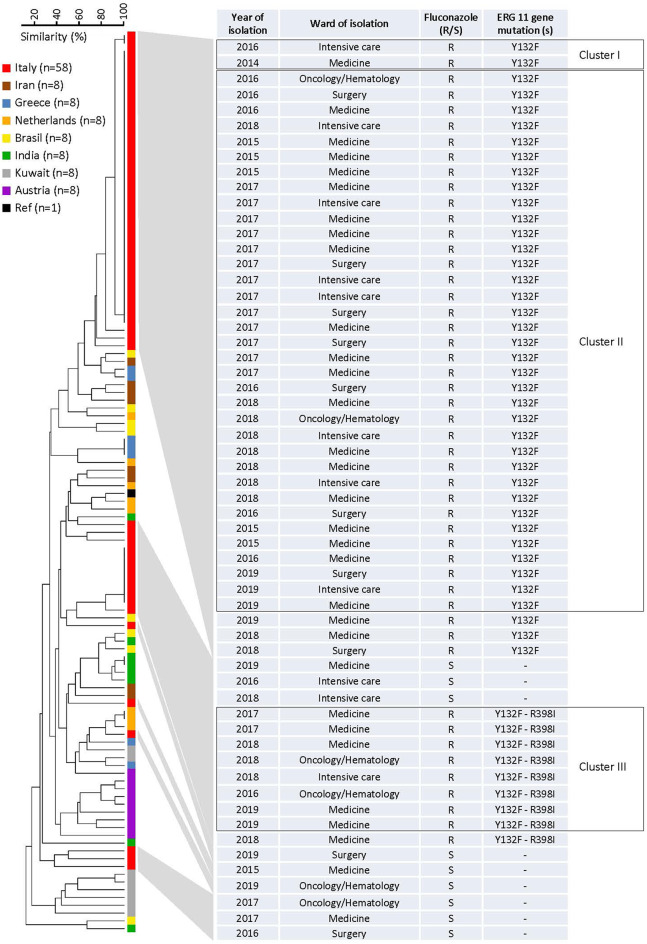
Dendrogram of similarity based on the microsatellite genotypes from 115 *Candida parapsilosis* isolates. The scale bar in the upper left corner indicates the similarity percentages, whereas the columns on the right report details on the 58 Italian isolates. Clusters I–III in the boxes denote those fluconazole-resistant isolates that harbored an amino acid substitution in the Erg11p, the azole-target enzyme encoded by the *ERG11* gene.

**Figure 3:**
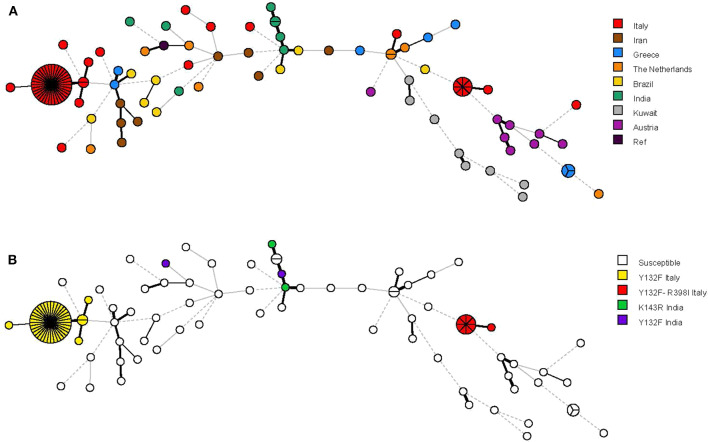
Minimum spanning tree analysis based on the microsatellite genotypes from 115 *Candida parapsilosis* isolates. Each circle corresponds to a unique genotype, whereas the sizes of the circles correspond to the numbers of isolates in the same genotype. The lines between the circles represent relative distance between isolates. The colors of the circles represent in **(A)** isolates from different hospitals and in **(B)** isolates harboring or not harboring the Erg11p Y132F (alone or in combination with the Erg11p R398I) substitution.

The relationship between the 68 microsatellite genotypes is depicted in Figures 2, 3. The dendrogram shown in Figure 2 illustrates two major clusters consisting of fluconazole-resistant isolates (43/49). While all isolates from cluster II harbored the Y132F substitution, those from cluster III also harbored the R398I substitution. Interestingly, the other Italian isolates with Y132F (cluster I, 2 isolates) or Y132F-R398I (one isolate) substitutions were closely related to cluster II and III, respectively, while the two Indian isolates with the Y132F substitution had a clearly different genotype than the isolates from clusters II and I. As detailed in Figure 2, the main Italian clusters appeared in the hospital from 2015 (cluster II) or 2016 (cluster III) and persisted until 2019, tending to be most apparent in 2017 (cluster II, 13/35 isolates) or 2018 (cluster III, 3/8 isolates). Alike, most patients infected by the isolates of cluster II (19/35) or cluster III (5/8) were from medical wards.

The minimum spanning tree shown in Figure 3 illustrates the differences between the genotypes based on a categorical analysis. Among the isolates harboring the Y132F substitution (40 isolates) and the Y132F-R398I substitution (9 isolates), there were apparently closely related genotypes that differed in only one of the six microsatellite markers and comprised, respectively, 2 isolates (cluster I) and 1 isolate (single). Three other single genotypes differed in two of the six microsatellite markers.

## Discussion

This study led to some important observations. First, we analyzed a large collection of fluconazole-resistant isolates of *C. parapsilosis* that were causing BSI in patients from a single hospital in Italy. These isolates represented almost the totality of fluconazole-resistant *C. parapsilosis* BSI episodes recorded by us between May 2014 and May 2019 and we excluded only four episodes from our analysis due to unavailability of isolates. We noted that the frequency of *C. parapsilosis* isolates found to be non-susceptible (resistant and SDD) to fluconazole did vary in the study periods (4/34 < 8/45 < 12/42 < 19/58 > 11/62 isolates), reaching the highest from May 2017 to May 2018. However, significance of this data may be underpowered because of relatively low numbers, as well as it is difficult to explain why the frequency of non-susceptible isolates decreased from May 2018 to May 2019. While *C. parapsilosis* lately became the second cause of candidemia in European centers, it yet remains unclear how the concomitant conditions of the patients, the interventions they undergo, and the drugs used may have concurred with the emergence of *C. parapsilosis* as a bloodstream pathogen (Silva et al., 2012). Until recently, a population-based surveillance study conducted in Barcelona (Spain) showed being a hematologic transplant, having prior fluconazole exposure, and being of a neonatal age as independent predictors of *C. parapsilosis* (and other NCAC) BSI (Rodríguez et al., 2010). In our study, the majority of *C. parapsilosis* BSI episodes identified (i.e., either due to resistant or susceptible isolates) occurred in adult patients (mainly from medical wards), and only a minority occurred in pediatric or neonatal patients. The reasons for these apparently conflicting data are unknown, but it is plausible that a diversity in antifungal prophylaxis practices or overall incidences among patient populations might explain the differences between the two studies. Altogether, these observations are consistent with traits found in *C. parapsilosis* infections (i.e., candidemia and other forms of candidiasis), such as nosocomial outbreaks, horizontal transmission, and resistance to azole antifungal agents (Pfaller and Castanheira, 2016). While such species-specific traits complicate our understanding of what makes *C. parapsilosis* a serious problem, they may actually hinder the eradication of infection by and the efficacy of antifungal therapy for hospitalized patients (Tóth et al., 2019).

Although triazoles continue to be useful for treating *C. parapsilosis* infections (Pristov and Ghannoum, 2019), we as well as other mycologists (Ostrosky-Zeichner and Andes, 2017) chose to routinely perform the testing of azole susceptibility for all *Candida* isolates associated with invasive disease (i.e., all bloodstream and other sterile site isolates). Adopting this strategy allows to monitor the trends of resistance over time and to track the emergence of isolates with an elevated MIC for a given antifungal agent. Compared to the study of antifungal susceptibility profiles of bloodstream yeast isolates during the years from 2005 to 2013 (Posteraro et al., 2015), we noted that in the present study, the rates of *C. parapsilosis* susceptibility (WT phenotype) to fluconazole and voriconazole decreased over the years from 92.3 to 77.6%. Therefore, we thought there was value in assessing the resistance mechanisms via genotypic studies, as this (from the laboratory perspective) is key to detecting those molecular changes responsible for the elevated MIC phenotype, and importantly, the reduced antifungal effect.

We characterized azole resistance mechanisms of 49 *C. parapsilosis* isolates with NWT phenotypes to fluconazole and voriconazole. We found that all isolates had the Y132F substitution in Erg11p, indicating that the alteration on position 132 in the lanosterol 14-α-demethylase target enzyme may be mainly responsible for azole resistance in *C. parapsilosis*. In line with the findings from studies conducted in the United States (Berkow et al., 2015; Grossman et al., 2015), we detected the Erg11p Y132F substitution in combination with the Erg11p R398I substitution, but only for 9 of 49 isolates. The R398I substitution is unable to confer azole resistance on its own and seems to be rather a compensatory mutation (Berkow and Lockhart, 2017). Among the fluconazole and voriconazole NWT isolates of *C. parapsilosis* collected worldwide during 2016–2017 by Castanheira et al. (2019), 37 (80.4%) of 46 isolates harbored the Y132F substitution and 35 of these isolates (18 in 2016 and 17 in 2017) were from three Italian hospitals, 22 of which were from a single hospital in Genoa. Of note, a recently conducted study in Italy provided the first evidence that the Y132F substitution (i.e., the A395T mutation in the *ERG11* gene) confers also multi-azole resistance in clinical isolates of *C. orthopsilosis* (Rizzato et al., 2018). Besides altering the binding site of the enzyme—thus preventing the binding of azoles—mutation in *ERG11* prompts the fungal cell to develop bypass pathways (i.e., not interrupted by azoles) to maintain a functional cell membrane (Pristov and Ghannoum, 2019). Thus, alterations in the ergosterol biosynthetic pathway—together with efflux-pump gene alterations—are central to the development of azole resistance in *C. parapsilosis* (Tóth et al., 2019). Interestingly, resistance to azoles and echinocandins—*C. parapsilosis* has a natural polymorphism in the *FKS1* gene, resulting in a decreased sensitivity of the corresponding glucan synthase to echinocandins—may be linked to single pathways (Tóth et al., 2019). Recently, a G111R substitution in the sterol desaturase encoding *ERG3* gene was responsible for altered azole and echinocandin susceptibilities in *C. parapsilosis* (Rybak et al., 2017). Nonetheless, the SENTRY antifungal surveillance program from 1997 to 2016 documented the sustained activities of fluconazole (azoles) and echinocandins against invasive isolates of *Candida* species, confirming that fluconazole-resistant and echinocandin-resistant *C. parapsilosis* isolates remain uncommon (Pfaller et al., 2019).

The emergence of clonal (and non-clonal) resistance in clinical, or hospital, settings worldwide is of great concern (Ostrosky-Zeichner and Andes, 2017). An example is *Candida auris*, a formidable yeast just owing to its multidrug resistance (Pristov and Ghannoum, 2019). While the high clonal inter- and intra-hospital transmission implies *C. auris* to become widespread across several countries (de Groot et al., 2020), it is noteworthy that the Erg11p Y132F substitution has also been reported in *C. auris* isolates from India, Pakistan, and Venezuela, and that these isolates are strongly associated with clonal transmission (Lockhart et al., 2017; Chowdhary et al., 2018). However, the Y132F substitution encompassed all isolates as in our study (100%) or only a part, albeit considerable, as reported elsewhere—i.e., 31.0–51.0% (Berkow et al., 2015; Grossman et al., 2015; Souza et al., 2015; Asadzadeh et al., 2017) or 63.8% (Choi et al., 2018)—of fluconazole-resistant *C. parapsilosis* isolates. Therefore it is important, when feasible, to consider these findings together with those of genotyping analyses that demonstrate a low genetic diversity among Y132F isolates. Choi et al. (2018) used microsatellite analysis to reveal that the proportion of clonal *C. parapsilosis* isolates was much higher in the group of fluconazole-resistant isolates harboring the Y132F substitution (86.7%, 26/30) than in the group of fluconazole-resistant isolates not harboring the Y132F substitution (11.8%, 2/17). Of note, all the 30 isolates represented four clonal isolates—based on a genotype resulting from combination of the sizes of four markers—and persisted within two South Korea hospitals for several years (Choi et al., 2018). Clonal isolates in that study were ≥2 isolates with identical genotypes according to microsatellite typing.

The results of microsatellite analysis in our study were consistent with those of few dominant genotypes identified by Choi et al. (2018). The high mutation rate of microsatellites—multiple 2- to 10-bp STR—makes this analysis particularly suited to detect micro-evolutionary variations of isolates obtained from different patient's body sites, and also, from nosocomial outbreaks involving different patients (van Asbeck et al., 2009). However, unlike Choi et al. (2018), we incorporated a greater number of markers in our microsatellite analysis to improve its discriminatory power. Therefore, according to a method developed (Sabino et al., 2010) and adopted (Diab-Elschahawi et al., 2012) to distinguish among *C. parapsilosis* isolates, we used a six-marker microsatellite panel for the present molecular investigation. Our isolates were collected only from Italian patients, and it was intuitive that coevolution of genetic markers could provide similar results by any selected typing method. So, we did not use a multilocus sequence typing or an automated repetitive sequence-based PCR as an additional genotyping method (Diab-Elschahawi et al., 2012) but, to strengthen our molecular investigation, we included random control isolates obtained from patients of seven countries other than Italy (Austria, Brazil, Greece, India, Iran, Kuwait, and the Netherlands).

Of 68 different genotypes identified in our study, two dominant genotypes were observed in 35 and eight isolates, respectively, which resulted in two clusters covering only the isolates from Italy and, importantly, 43 (87.7%) of 49 isolates cross-resistant/NWT to fluconazole and voriconazole (all harboring the Y132F substitution). Another genotype was observed in two (4.1%) of 49 isolates. Four other genotypes were observed only once. Because of microevolution due to intrinsic instability of microsatellite loci, we considered the isolates with only minor changes in one of the microsatellite markers as clonally related though not identical. This may suggest the existence of clonal complexes that exclusively contained all the 49 fluconazole-resistant Italian isolates. Conversely, all 9 fluconazole-susceptible Italian isolates as well as 56 non-Italian isolates belonged to unique genotypes, which were different from the cluster genotypes. As microsatellite genotypes may acquire resistance through azole exposure at differing frequencies (Hou et al., 2017), the hypothesis of a link between genotype and susceptibility to fluconazole (and other triazoles) is highly verisimilar, as already suggested for *C. parapsilosis* (Desnos-Ollivier et al., 2018) and other NCAC species (Hou et al., 2017). Accordingly, no studies to date found an association between prior antifungal exposure and fluconazole-resistant *C. parapsilosis* BSIs (Govender et al., 2016; Choi et al., 2018), whereas our registration of prior antifungal exposure was, unfortunately, incomplete (data not shown). Thus, it is possible that azole-resistant isolates (in particular, Y132F isolates) emerged following the overuse of triazoles for prophylaxis and treatment of invasive *Candida* and other fungal infections, and subsequently transmitted via patient-to patient contact. Two of our patients suffered from subsequent BSIs with two genotypically identical isolates each—that both belonged to cluster II in one patient and to cluster III in the other patient—suggesting an endogenous source of their infections (Brillowska-Dabrowska et al., 2010). If the dissemination of Y132F isolates is due to the propensity to survive outside their host and, hence, in the hospital environment—perhaps coupled to poor healthcare workers' adherence to standard contact precautions—this needs to be determined.

This study has some limitations. As in other NCAC species, the mechanisms contributing to azole resistance in *C. parapsilosis* isolates may be multiple (Pristov and Ghannoum, 2019). Although an alteration in *ERG11* leads to a modification in the binding site of the enzyme targeted by the azoles, this alteration can be through either point mutations or upregulation. However, only one among the *C. parapsilosis* isolates from a global antifungal surveillance multicenter study (Castanheira et al., 2019) overexpressed the *ERG11* gene. By contrast, in the same study, most of the Italian Y132F isolates overexpressed the *MDR1* gene, which encodes for a major-facilitator superfamily efflux pump (Pristov and Ghannoum, 2019). Thus, we did not rule out that additional azole resistance mechanisms could have contributed to the MIC phenotypes observed in our isolates. Furthermore, we did not explore transcription factors such as *MRR1*, which can lead to the upregulation of *MDR1*, or *UPC2*, which can lead to the upregulation of *ERG11* (Pristov and Ghannoum, 2019). However, all but one of the Y132F isolates from South Korea had the K177N substitution in Mrr1p, whereas none of the isolates had a missense mutation in Upc2p (Choi et al., 2018). In practice, our decision of not extending molecular resistance analyses relies on the undisputable finding that *C. parapsilosis ERG11* alleles carrying Y132F or K143R substitutions were able to confer elevated azole MICs (≥16-fold) when expressed in azole-susceptible *Saccharomyces cerevisiae*, as shown recently (Singh et al., 2019). Finally, the other aspect that the Y132F (and K143R) substitutions in clonal isolates may enable these isolates to acquire a potential for intra-hospital persistence and/or dissemination also warrants further studies.

## Conclusion

Fluconazole-resistant clonal isolates of *C. parapsilosis* harboring the Y132F substitution were circulating in our hospital. While this molecular resistance mechanism was apparently dominant, the clonal lineage of Y132F isolates (i.e., two main clusters of isolates with identical genotypes that infected different patients) could indicate either cross-infection among patients or direct infection of patients—the latter through a possible common source—in our hospital. We confirmed the microsatellite-based typing method as a helpful tool to answer questions of isolate relatedness. Therefore, detecting the Y132F (and K143R) substitutions in any bloodstream *C. parapsilosis* isolate recovered routinely by means of a rapid molecular assay could be an attractive way to identify clonal *C. parapsilosis* isolates in hospital or clinical settings and, ultimately, prevent their transmission among patients at risk of BSI.

## Data Availability Statement

The raw data supporting the conclusions of this article will be made available by the authors, without undue reservation, to any qualified researcher.

## Ethics Statement

The study did not require oversight by the institutional ethics committee because the descriptive nature implied only samples that were obtained during routine laboratory activity.

## Author Contributions

CM, BP, JM, and MS conceived and designed the experiments. CM, RT, and TG performed the experiments. CM, RT, TG, ED, GM, GD, BP, JM, and MS analyzed the data. CM and BP wrote the paper. All the authors read, improved, and approved the final version of the manuscript.

## Conflict of Interest

The authors declare that the research was conducted in the absence of any commercial or financial relationships that could be construed as a potential conflict of interest.

## Supplementary Material

Table S1 Detailed information on the 58 Italian and 56 non-Italian (control) isolates studied. This included data on the geographical origin, year of isolation, fluconazole susceptibility phenotype, and microsatellite genotype for all the isolates. Other details, whenever available, were shown.
